# Giant retinal pigment epithelial tear following photodynamic therapy for the bullous variant of central serous chorioretinopathy: A case report

**DOI:** 10.1097/MD.0000000000037855

**Published:** 2024-04-19

**Authors:** Eri Kimura, Masahiro Miura

**Affiliations:** a Department of Ophthalmology, Tokyo Medical University, Ibaraki Medical Center, Inashiki, Ibaraki, Japan.

**Keywords:** bullous variant of central serous chorioretinopathy, case report, photodynamic therapy, retinal pigment epithelium tear

## Abstract

**Rationale:**

The bullous variant of central serous chorioretinopathy (CSC) is a severe form of chronic CSC. Patients with the bullous variant of CSC have an increased risk of experiencing multiple pigment epithelial detachments (PEDs) and retinal pigment epithelium (RPE) tears. Photodynamic therapy (PDT) is a treatment for the bullous variant of CSC. RPE tear is a possible postoperative complication of PDT for eyes with PEDs. To our knowledge, no cases of giant RPE tears following PDT for the bullous variant of CSC have been reported previously. This case report presents the first instance of a giant RPE tear after half-time PDT for the bullous variant of CSC, accompanied by a series of images depicting the tear development.

**Patient concerns:**

A 63-year-old male patient presented with rapidly deteriorating vision in his left eye over a 3-month period. He also reported a previous episode of vision loss in his right eye 2 years prior. Best-corrected visual acuity (BCVA) in the left eye was 0.2.

**Diagnosis:**

The right eye was diagnosed with chronic non-bullous CSC, while the left eye was diagnosed with the bullous variant of CSC with a large PED.

**Interventions:**

Half-time PDT was administered to the left eye.

**Outcomes:**

One month after half-time PDT, a giant RPE tear exceeding 3 clock-hours in size was confirmed in the lower temporal quadrant of the left eye. Three months after the initial half-time PDT, a second half-time PDT was performed owing to recurrent retinal detachment. Two months after the second half-time PDT, the retinal detachment resolved, and BCVA improved to 0.4, 6 months after the second half-time PDT.

**Lessons:**

In cases where the bullous variant of CSC is complicated by extensive PED, clinicians should consider the potential development of a giant RPE tear as a treatment complication.

## 1. Introduction

The bullous variant of central serous chorioretinopathy (CSC) is a rare and severe form of chronic CSC, characterized by multifocal fluid leakage, bullous retinal detachment, and shifting subretinal fluid.^[1–3]^ Compared with patients with non-bullous chronic CSC, patients with the bullous variant of CSC are more prone to developing multiple pigment epithelial detachments (PEDs) and retinal pigment epithelium (RPE) tears.^[2–4]^ Various treatment options have been explored for the bullous variant of CSC, including photodynamic therapy (PDT), focal photocoagulation, intravitreal anti-vascular endothelial growth factor injections, corticosteroid withdrawal, and vitrectomy.^[1,3–5]^ A recent study found that PDT is the most common initial treatment for the bullous variant of CSC.^[3]^ However, it is crucial to acknowledge the potential for RPE tears as a serious complication of PDT in eyes with PEDs.^[6,7]^ This case report presents a unique instance of the bullous variant of CSC with a large PED complicated by a giant RPE tear following PDT.

## 2. Ethics statement

Ethical approval for this study was obtained from the Institutional Review Board of Tokyo Medical University (approval number: T2020-142). The study protocol adhered to the tenets of the Declaration of Helsinki. Written informed consent for the publication of this case report, including the accompanying images, was obtained from the patient after providing a detailed explanation of the report and ensuring his full understanding.

## 3. Case

A 63-year-old male patient presented to our clinic with rapidly progressive vision loss in his left eye over a duration of 3 months. He also reported a previous episode of vision loss in his right eye 2 years earlier. His best-corrected visual acuity (BCVA) was 0.1 in the right eye and 0.2 in the left eye. He had no history of retinal disease or systemic conditions with retinal involvement. Additionally, his family history was negative for ocular or hereditary diseases.

**Figure 1.**
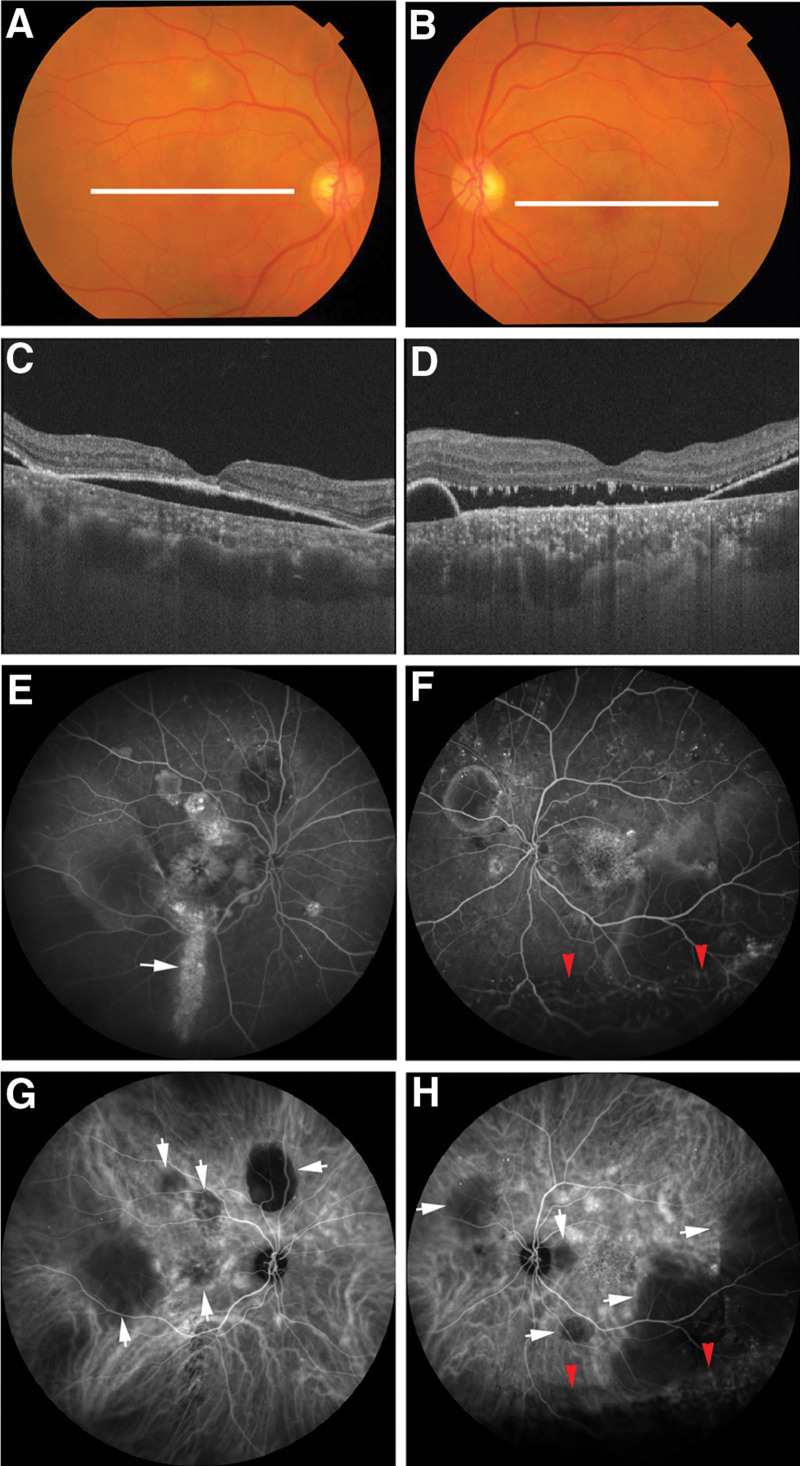
Multimodal imaging findings before half-time photodynamic therapy. Color fundus photograph of the right (A) and left (B) eyes showing white lines indicating the scanning line of the optical coherence tomography (OCT) images of the right and left eyes, respectively. OCT image of the right eye (C) showing pigment epithelial detachment (PED) in the macula. OCT image of the left eye (D) showing retinal detachment with PED. Fluorescein angiography image of the right eye (E) showing multiple leakages and hyperfluorescence at the descending retinal pigment epithelium (RPE) tracking lesion (white arrow). Fluorescein angiography image of the left eye (F) showing multiple leakages. The area of bullous retinal detachment shows hypofluorescence (red arrowheads). Indocyanine green angiography (ICGA) image of the right (G) and left (H) eyes showing multiple hyperfluorescent areas suggestive of choroidal hyperpermeability. In the early phase of ICGA, the area of the PED showed hypofluorescence in both eyes (white arrows), and the area of bullous retinal detachment showed hypofluorescence in the left eye (red arrowheads).

On fundus examination of the right eye, multiple PEDs were observed, and optical coherence tomography (OCT) revealed a PED without retinal detachment in the macula (Fig. 1A and C). In the left eye, fundus examination revealed bullous inferior retinal detachment with shifting subretinal fluid. Multiple PEDs were also observed, including a large PED that exceeded 3 clock-hours in size in the lower temporal quadrant. OCT confirmed retinal detachment in the macula (Fig. 1B and D). Ultrawide field retinal angiography was performed using a scanning laser ophthalmoscope (HRA2; Heidelberg Engineering, Heidelberg, Germany). Fluorescein angiography showed multiple leakages involving the macula in both eyes (Fig. 1E and F). The right eye displayed hyperfluorescence at the descending atrophic RPE tracking lesion. In the left eye, the area of bullous retinal detachment showed hypofluorescence. Indocyanine green angiography revealed multiple hyperfluorescent areas suggestive of choroidal hyperpermeability in both eyes (Fig. 1G and H). In the early phase, areas of PEDs exhibited hypofluorescence in both eyes. The area of bullous retinal detachment in the left eye showed hypofluorescence. On the basis of the overall findings in these images, the right eye was diagnosed with chronic non-bullous CSC, and the left eye was diagnosed with the bullous variant of CSC.

Owing to the patient rapid left eye vision loss and in accordance with his request, half-time PDT was performed on the left eye. Half-time PDT was administered using 6 mg/m² verteporfin (Visudyne; QLT Inc., Vancouver, BC, Canada) and a 7.0-mm laser spot size for 42 seconds, with an irradiance of 600 mW/cm².^[8]^ The treatment was directed at the central macula, including the fovea.

**Figure 2.**
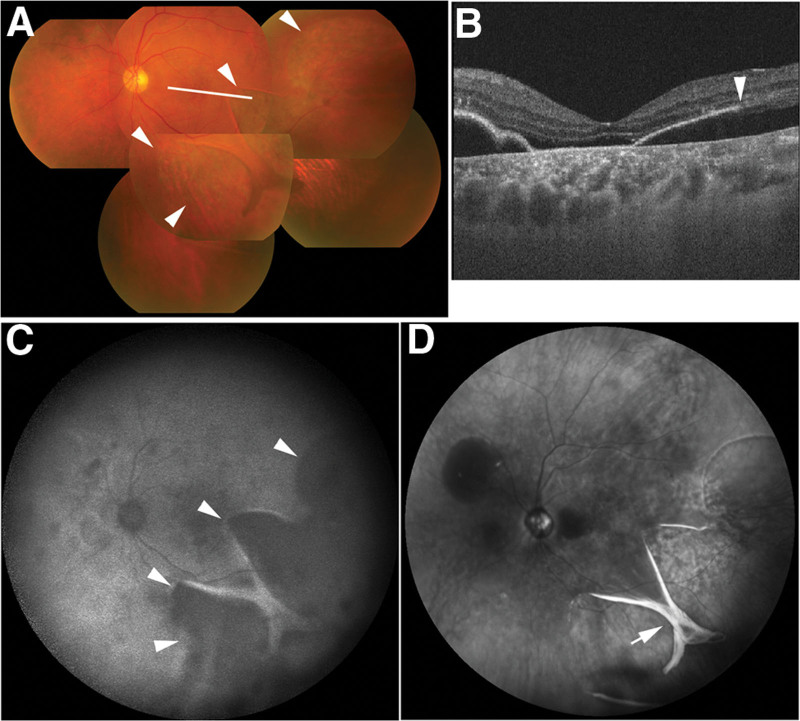
Multimodal imaging findings of the left eye 1 mo after half-time photodynamic therapy. Color fundus photograph (A) showing a giant retinal pigment epithelium (RPE) tear exceeding 3 clock-hours in size (white arrowheads). The white line indicates the scanning line of the optical coherence tomography (OCT) image. OCT image (B) showing the edge of the giant RPE tear (white arrowhead). Autofluorescence imaging (C) demonstrating hypoautofluorescence in the area of the giant RPE tear (white arrowheads). Near-infrared image (D) showing a rolled edge in the giant RPE tear (white arrow).

One month after half-time PDT, the patient presented for follow-up, and the BCVA in his left eye had decreased to counting fingers. Fundus examination revealed a giant RPE tear exceeding 3 clock-hours in size in the lower temporal quadrant (Fig. 2A). OCT showed a reduction in retinal detachment and the edge of the RPE tear on PED (Fig. 2B). In ultrawide field autofluorescence imaging (excitation: 488 nm) obtained with the HRA2, the area of the giant RPE tear exhibited hypoautofluorescence (Fig. 2C). Ultrawide field near-infrared imaging at 820 nm obtained with the HRA2 demonstrated a rolled edge in the giant RPE tear (Fig. 2D).

**Figure 3.**
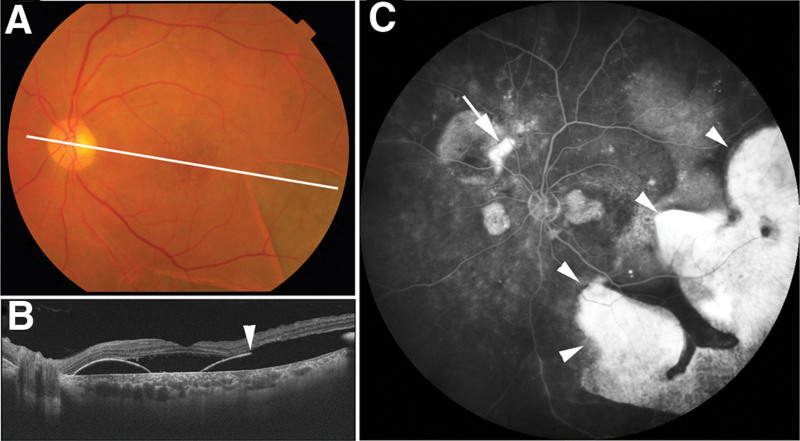
Multimodal imaging findings of the left eye 3 mo after half-time photodynamic therapy. Color fundus photograph (A) showing a white line indicating the scanning line of the optical coherence tomography (OCT) image. OCT image (B) showing retinal detachment with pigment epithelial detachment (PED). The white arrowhead indicates the edge of a giant retinal pigment epithelium (RPE) tear. Fluorescein angiography image (C) showing hyperfluorescence in the area of the RPE tear due to window defects (white arrowheads). An additional RPE tear is also visible superior-nasal to the optic nerve head (white arrow).

**Figure 4.**
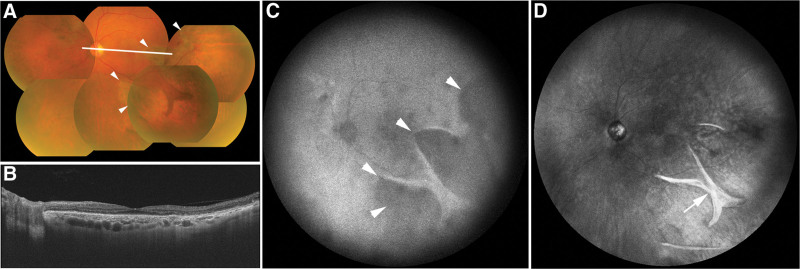
Multimodal imaging findings of the left eye 6 mo after the second half-time photodynamic therapy. Color fundus photograph (A) showing a persistent giant retinal pigment epithelium (RPE) tear (white arrowheads). The white line indicates the scanning line of the optical coherence tomography (OCT) image. OCT image (B) showing the absence of retinal detachment or pigment epithelial detachment. Autofluorescence image (C) showing no change in the extent of the RPE tear compared with that in the image obtained after the first photodynamic therapy (white arrowheads). Near-infrared image (D) confirming the presence of a rolled edge in the RPE tear at the same location as that in the image obtained after the first photodynamic therapy (white arrow).

Three months after PDT (Fig. 3A–C), the patient BCVA in the left eye remained at counting fingers, and OCT showed worsening of the macular retinal detachment (Fig. 3B). Fluorescein angiography revealed multiple leakages and hyperfluorescence at the giant RPE tear (Fig. 3C). An additional RPE tear was also confirmed superior-nasal to the optic nerve head. Despite the occurrence of a giant RPE tear after the first half-time PDT, we performed a second half-time PDT, assessing that the likelihood of another RPE tear during the second treatment was low, given that the large PED no longer existed following the occurrence of the giant RPE tear. Two months after the second half-time PDT, the macular retinal detachment resolved, and BCVA improved to 0.4, 6 months after the second half-time PDT (Fig. 4A and B). Autofluorescence images taken 6 months after the second half-time PDT showed that the extent of the RPE tear remained unchanged compared with the image obtained after the first PDT, and near-infrared imaging confirmed the presence of a rolled edge of the RPE tear in the same location (Fig. 4C and D).

## 4. Discussion

The bullous variant of CSC is a severe form of chronic CSC, first described by Gass in 1973.^[1]^ The case of the bullous variant of CSC reported here had an RPE tear that exceeded 3 clock-hours in size. Consistent with previous reports, we identified this as a giant RPE tear.^[9,10]^ To the best of our knowledge, this is the first documented instance of giant RPE tear formation following PDT for the bullous variant of CSC.

The development of this RPE tear is likely attributable to a combination of factors. First, RPE tear is an inherent feature of the natural course of the bullous variant of CSC.^[2,3]^ Second, RPE tear is a potential complication of PDT for CSC.^[7]^ Third, this particular case was characterized by the presence of a large PED. In cases of age-related macular degeneration, a large PED is a known risk factor for RPE tear.^[11]^ To summarize these considerations, RPE tear is an inherent feature of the bullous variant of CSC. Additionally, the presence of a large PED in this case is thought to have further increased the risk of an RPE tear following PDT.

Several causal mechanisms have been proposed to explain the etiology of RPE tear following PDT. One possibility is that PDT inflicts additional damage to the RPE,^[12]^ which is already compromised in the bullous variant of CSC.^[1–3]^ Another potential mechanism is that PDT-induced alterations in choroidal perfusion weaken RPE adhesion.^[13]^ Additionally, the accumulation of fluid within PEDs in the days following PDT may contribute to RPE tear development.^[14]^ These multifaceted factors may collectively contribute to the development of RPE tears after PDT in the bullous variant of CSC.

In this case, considering the rapid vision loss and the poor visual prognosis of the bullous variant of CSC,^[4]^ treatment was initiated immediately without observation. Half-time PDT was chosen as the treatment of choice in this case as PDT is generally considered a viable treatment option for the bullous variant of CSC,^[3]^ and the effectiveness of half-time PDT for CSC has been reported.^[8]^ Previous studies have explored various treatment approaches for the bullous variant of CSC.^[1,3–5]^ However, the bullous variant of CSC is a rare disease, limiting the number of cases reported in the literature. Consequently, an optimal treatment protocol has yet to be established. A case series found that the visual prognosis of the bullous variant of CSC is unaffected by whether the condition is treated.^[4]^ Another report suggested that the treatment response for the bullous variant of CSC is more favorable than that for chronic non-bullous CSC.^[3]^ Some researchers have hypothesized that the presence of RPE tears promotes the healing of CSC.^[3]^ Conversely, a poor visual prognosis of the bullous variant of CSC has been reported,^[4]^ and poor vision at diagnosis is associated with a worse visual outcome.^[3]^ To establish a comprehensive treatment strategy for the bullous variant of CSC, further research is warranted to increase the number of cases studied and provide a comprehensive evaluation.

This case report has several limitations. First, as mentioned, there is no consensus on the treatment strategies for the bullous variant of CSC. Second, this report involved a single case, which limits the generalizability of our findings. Third, the early implementation of half-time PDT in this case precludes any insights into the natural course of the disease. These limitations underscore the need for long-term studies with a large number of cases to fully elucidate the evolving clinical pathophysiology of the bullous variant of CSC.

In conclusion, we presented a case of giant RPE tear following half-time PDT for the bullous variant of CSC. The presence of a large PED likely contributed to the development of the giant RPE tear in this case. Additionally, a previous report documented an RPE tear involving the macula following photocoagulation treatment for chronic CSC with a PED involving the macula.^[15]^ Therefore, in cases of the bullous variant of CSC complicated by a large PED or a PED involving the macula, there is potential for the development of a giant RPE tear or an RPE tear of the macula as a treatment complication. Consequently, careful consideration and planning are essential when determining the treatment strategy.

## Acknowledgments

This study was supported by Grants-in-Aid for Scientific Research (21K09684) from the Japan Society for the Promotion of Science. We thank Jane Charbonneau, DVM, from Edanz (https://jp.edanz.com/ac) for editing a draft of this manuscript.

## Author contributions

**Conceptualization:** Eri Kimura, Masahiro Miura.

**Data curation:** Eri Kimura, Masahiro Miura.

**Formal analysis:** Eri Kimura, Masahiro Miura.

**Funding acquisition:** Masahiro Miura.

**Investigation:** Eri Kimura, Masahiro Miura.

**Methodology:** Eri Kimura, Masahiro Miura.

**Project administration:** Masahiro Miura.

**Supervision:** Masahiro Miura.

**Validation:** Eri Kimura, Masahiro Miura.

**Visualization:** Eri Kimura, Masahiro Miura.

**Writing – original draft:** Eri Kimura, Masahiro Miura.

**Writing – review & editing:** Eri Kimura, Masahiro Miura.
